# Analysis of infection rates and duration of short and long-term hemodialysis catheters in a teaching hospital

**DOI:** 10.1590/1677-5449.190142

**Published:** 2020-09-14

**Authors:** Seleno Glauber de Jesus-Silva, Jennifer dos Santos Oliveira, Karine Tobias França Ramos, Luciene Azevedo Morais, Melissa Andreia de Moraes Silva, Arturo Eduardo Krupa, Rodolfo Souza Cardoso

**Affiliations:** 1 Associação de Integração Social de Itajubá – AISI, Hospital de Clínicas de Itajubá – HCI, Departamento de Cirurgia Vascular e Endovascular, Itajubá, MG, Brasil.; 2 Faculdade de Medicina de Itajubá – FMIt, Itajubá, MG, Brasil.; 3 Associação de Integração Social de Itajubá – AISI, Hospital de Clínicas de Itajubá – HCI, Departamento de Nefrologia, Itajubá, MG, Brasil.

**Keywords:** renal dialysis, catheter-related infections, indwelling catheters

## Abstract

**Background:**

Short-term (ST) and long-term tunneled (LTT) central venous catheters for hemodialysis (CVCH) are critical for hemodialysis therapy. However, few studies have been conducted in Brazil to investigate the incidence of complications with these two types of catheters.

**Objectives:**

To analyze complications and duration of CVCH in a hemodialysis center at a teaching hospital.

**Methods:**

Single-center, longitudinal, and retrospective study of 115 consecutive patients undergoing hemodialysis catheter placement (67 ST and 48 LTT) over a 2-year period, analyzing overall survival, patency, loss of access, and incidence of complications.

**Results:**

Sixty percent of the patients were male and mean age was 62 years. The most common puncture site was the right internal jugular vein. Systemic arterial hypertension was present in 95% of cases. Median catheter in-place duration was 50 days (ST) vs. 112 days (LTT; p < 0.0001). There was no difference in overall survival. Incidence of catheter-related infection was higher in ST CVCH, with *Staphylococcus* sp. the microorganism most often found. The infection rate per 1000 days was higher in ST than in LTT catheters (16.7 events/1000 days vs. 7.0 events/1000 days). Low income was the only factor related to higher incidence of infection.

**Conclusions:**

The in-place duration of long-term catheters was significantly longer compared to short-term CVCH, but still below the values reported in the literature and without impact on overall survival. Low income was a factor associated with catheter infection.

## INTRODUCTION

Hemodialysis is a resource widely used in the treatment of end-stage chronic renal failure and requires several critical elements, one of which is the vascular access. Although, in general, the method maintains patients’ quality of life, it is associated with high rates of complications and admissions, and mortality rates are as high as 10 to 25% per year.^1^ Although the ideal access for long-term hemodialysis maintenance is an arteriovenous fistula (AVF) or a prosthetic graft fistula, in Brazil, central venous catheters are used for hemodialysis (CVCH) in a large proportion of patients, with a prevalence of up to 20.5%.^2^ Use of CVCH has increased in line with the aging population and the growing number of patients starting hemodialysis with few options for creation of an AVF.

Non-tunneled, short-term catheters (STC) are a reliable option for scenarios in which it is necessary to immediately institute renal substitution therapy in the absence of definitive access. However, they should be removed as soon as possible because of the high risk of infectious complications.^3^ In cases in which creation of an AVF is impossible, whether because there is no adequate vein available or because of clinical fragility, long-term tunneled catheters (LTTC) are considered a longer-lasting option associated with fewer complications.^4^ Use of an STC for more than 3 months is used as a negative quality indicator at hemodialysis services in Brazil.^5^

A diverse range of complications are related to CVCH, including those related to insertion (hematoma, pseudoaneurysm, pneumothorax), catheter-related and central vein thrombosis, and, the most severe of all, catheter-related infection. This last is associated with elevated hospital admissions, expenditure, and mortality.^6^ Several factors have been associated with loss of CVCH, including advanced age, history of multiple previous accesses, educational level, quality of manipulation of the device by the nursing team, diabetes, immunodepression, and others.^7^ ^,^ ^8^

While the international literature offers a large body of knowledge on CVCH progression, few Brazilian studies have investigated in-place duration and complication rates. Therefore, it is necessary to study patient samples that can show whether the real-life results at Brazilian hemodialysis centers are comparable with those predicted in the literature, to diagnose and correct possible failings. The objective of this study was to analyze, in a teaching hospital hemodialysis center, the different outcomes of STC and LTTC with respect to durability, infectious and non-infectious complications, and the risk factors associated with them.

## METHODS

A retrospective, observational, longitudinal study was conducted, reviewing and analyzing the medical records for 115 consecutive patients who had had a CVCH fitted (67 STC and 48 LTTC) at a renal replacement therapy service at a quaternary teaching hospital between January 2016 and January 2018. The study was approved by the Itajubá Medical School Research Ethics Committee, under ruling number 2.170.323.

The sample size calculation was based on the estimated difference in CVCH survival at 30 days (60% for STC and 90% for LTTC), a test power of 80%, and a significance level of 95%, resulting in a sample size of 69 individuals per group. Since it was not possible to recruit enough patients for the LTTC group within the study period, the sample was considered to be selected by convenience.

STC were defined as non-tunneled central venous hemodialysis catheters, irrespective of the duration of use (models used: Arrow-Howes®LargeBore 12 Fr, 16 and 20 cm, Teleflex, Morrisville, North Carolina, United States; and Duo-Flow®Side x Side 12 Fr, 15 and 20 cm, Medcomp, Harleysville, Pennsylvania, United States). LTTC were defined as double-lumen tunneled catheters (model: Hemo-Cath® LT 12.5 Fr, 28 and 32 cm, Medcomp). STCs were inserted by a nephrologist or a vascular surgeon in a procedures room without fluoroscopy. Only STCs inserted by the surgeon were guided with ultrasound, while the nephrologists used the conventional anatomic landmarks technique. All LTTCs were inserted by a vascular surgeon in an angiography suite, using a conventional aseptic technique and ultrasound guidance. No preoperative antibiotics were administered to prevent access infections, following the institutional protocol. However, all of the patients were either on platelet anti-aggregation (100 mg/day of acetylsalicylic acid or 75 mg/day of clopidogrel bisulfate) or oral anticoagulation. There was no way of tracking possible failures to comply with anti-aggregation or anticoagulation throughout follow-up. Data were extracted from a dedicated database maintained by the hemodialysis service (NefroSys®, Porto Alegre, RS) and imported to an electronic spreadsheet. A multidisciplinary team continuously updated the electronic database. The study included all patients who had a short or long-term catheter fitted for whom complete medical records were available, excluding patients with catheters inserted via the femoral vein. Only the first placement of each type of catheter was considered for the study. Patients whose STC were substituted for LTTC during the study period were included in the catheter duration analysis twice. Only cases in which STC was the only type of access used were included in the global survival analysis. Technical failures of catheter placement that prevented them from being used were not counted as completed accesses and were therefore not eligible for inclusion in the study.

Data were collected on epidemiological variables (age, sex, race, educational level, family income, and healthcare provider), dates of catheter insertion and removal, the reason for removal, complications, risk factors (systemic arterial hypertension [SAH], diabetes mellitus, dyslipidemia, and smoking), infections and pathogens identified, and dates of deaths. SAH was defined as arterial blood pressure exceeding 140 × 90 mm Hg or use of hypotensives. Diabetes mellitus was defined as fasting glycemia > 126 mg/dL or postprandial glycemia > 200 mg/dL or continuous use of hypoglycemics. Dyslipidemia was defined as LDL > 130 mg/dL or use of statins. Smoking was defined as any continuous use of at least one cigarette per day. Data were analyzed on aerobic cultures of samples from the catheter tip, sectioned at the time of removal in a sterile medium, with results defined as positive if more than 100 CFU/mm^2^ were isolated.

Descriptive statistics calculated were means or medians and percentages. Inferential statistics used were the two-tailed *t* test for independent samples, the chi-square test (*Χ* ^2^) or Fisher’s test, depending on the variables being analyzed and after identifying outliers using the ROUT method. Comparison of catheter duration rates was performed using Kaplan-Meier survival curves, with log-rank test. The overall infection rate per 1,000 catheter days and the mortality rate for 1,000 patients/year were calculated. GraphPad Prism v.8 (San Diego, CA, USA) statistical software was used, and the statistical significance cutoff adopted was p < 0.05, with a 95% confidence interval.

## RESULTS

**Figure 1:**
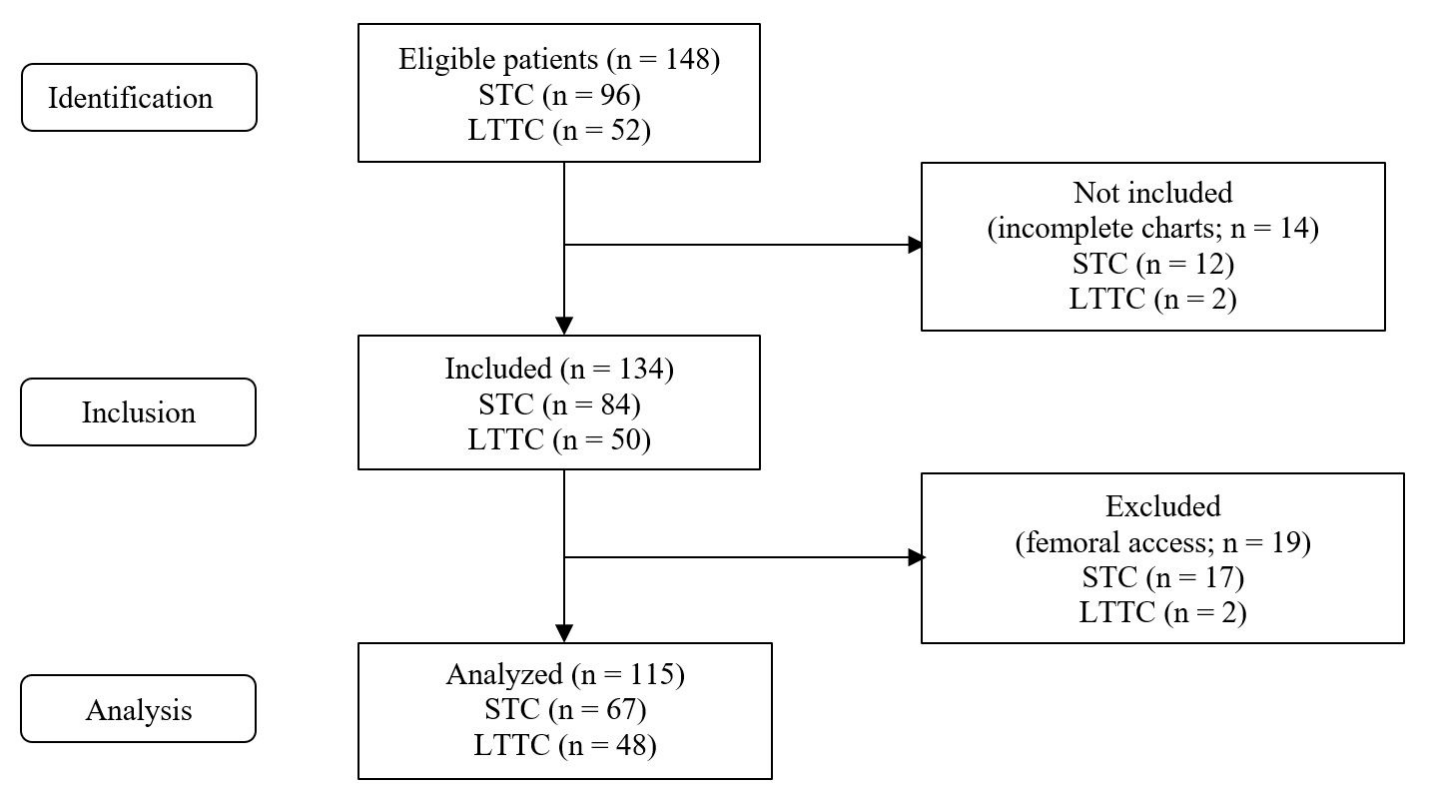
Flow diagram of patient selection. STC(short-term catheters); LTTC (long-term tunneled catheters).

One hundred and fifteen patients were analyzed, 67 of whom had an STC inserted, and 48 an LTTC (Figure 1). Sixty percent were male, and the mean age was 62 years. The right internal jugular vein was the most common puncture site, used for 85.1% of the STC and 79.2% of the LTTC. There were very high prevalence rates of SAH (95.7%) and diabetes mellitus (47%), while dyslipidemia (23.5%) and smoking (12.2%) were less frequent. There was no difference between groups in terms of body mass index (BMI). Twenty of 67 STC cases (29.9%) were inserted by a vascular surgeon. Table 1 shows the clinical characteristics and frequencies of access sites for both groups. All patients in the study were followed-up until the removal of the catheter or death.

**Table 1:** Epidemiological characteristics and risk factors in each of the subsets of the sample.

Risk factors	Total n (%)	STC n (%)	LTTC n (%)	p
Age (years)	62.2 (±15.0)^*^	58.2 (±14.5)^*^	67.8 (±13.9)^*^	0.0006^§^
Sex				
Male	69 (60)	44 (63.8)	25 (36.2)	0.14^£^
Female	46 (40)	23 (50)	23 (50)	
BMI	24 (±5.6)^*^	24.2 (±5.8)^*^	23.7 (±5.4)^*^	0.69 ^§^
Comorbidities				
Hypertension	110 (95.7)	63 (94)	47 (98)	0.39^†^
Diabetes mellitus	54 (47)	30 (45)	24 (50)	0.58^£^
Dyslipidemia	27 (23.5)	9 (13.4)	18 (37.5)	0.003^†^
Smoking	14 (12.2)	11 (16.4)	3 (6.3)	0.14^†^
Type of access				0.18^£^
Femoral	4 (3.5)	3 (4.5)	1 (2.1)	
Jugular D	95 (82.6)	57 (85.1)	38 (79.2)	
Jugular E	14 (12.2)	5 (7.5)	9 (18.8)	
AxV	2 (1.7)	2 (3.0)	0 (0)	

STC = non-tunneled short-term catheter; LTTC= tunneled long-term catheter; BMI = body mass index; AxV = axillary vein;

* standard deviation;

§ Student’s *t* test;

^£^Chi-square;

† Fisher’s exact test.

**Figure 2:**
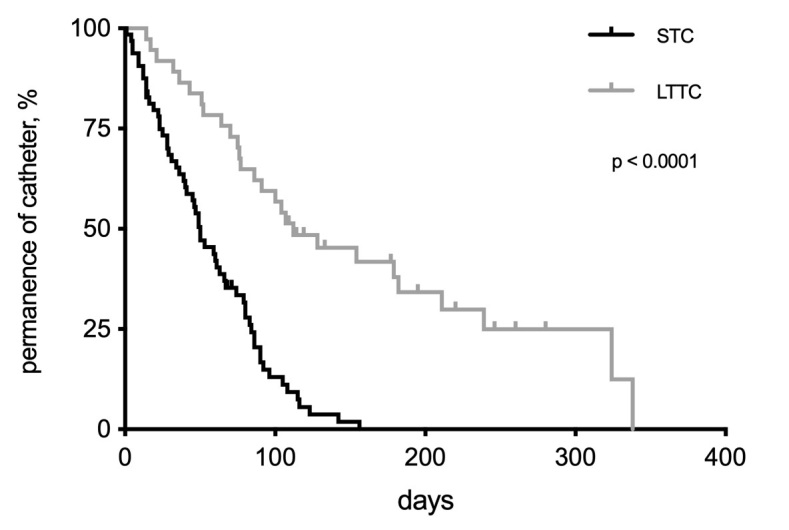
Kaplan-Meier curves for non-tunneled short-term catheters (STC) and long-term tunneled catheters (LTTC) inserted during a maximum period of 338 days of observation. A significant difference was detected between the groups (log-rank test).

**Figure 3:**
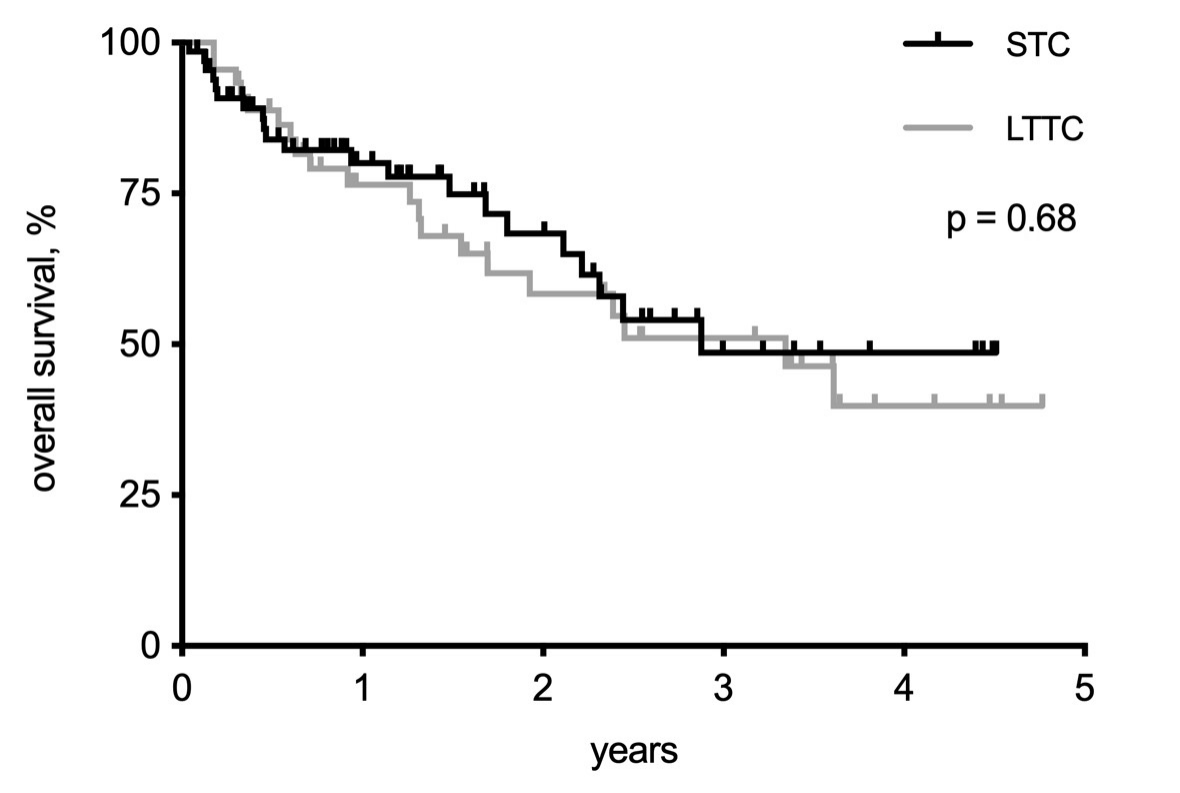
Kaplan-Meier curves for overall survival of patients with short-term catheters (STC) and long-term tunneled catheters (LTTC) over 4.8 years of observation. No significant difference was observed between the groups (log-rank test).

The in-place duration of the two catheter types was significantly different, with a median of 50 days for STC and 112 days for LTTC (95% confidence interval [CI], STC: 45.1-63.3 days vs. LTTC: 101.7-159.7 days; log-rank, p < 0.0001) (Figure 2). There were 21 deaths in the STC group and 20 deaths in the LTTC group during the entire follow-up period. Operative mortality (30 days) was 1.5% in the STC group, and there were no deaths in the LTTC group during the same period. The survival analysis did not detect a difference in overall mortality between groups (median survival STC: 2.87 years vs. LTTC: 3.34 years; log-rank, p = 0.68), and total mortality in the first year was 20% for STC and 23.5% for LTTC (Figure 3).

**Figure 4:**
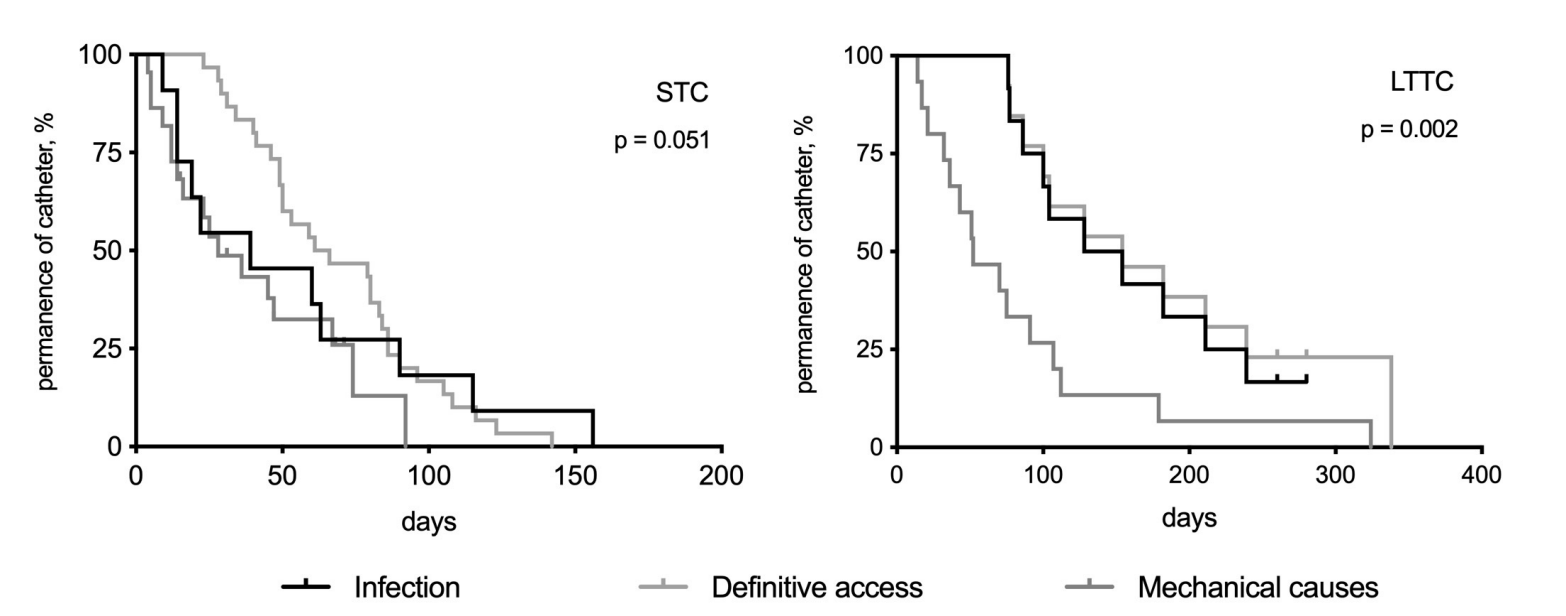
Kaplan-Meier curves for catheter survival based on the cause of removal (catheter infection; creation of definitive access, whether arteriovenous fistula or insertion of tunneled long-term catheter [LTTC]; or mechanical causes such as thrombosis, kinking, or dislodgement). In the STC group (non-tunneled short-term catheters), no significant difference was observed, but definitive access was the least common cause of removal. In the LTTC group, mechanical causes were the most common reason for catheter withdrawal or exchange.

Causes of catheter removal were classified as definitive access (creation of an arteriovenous fistula or LTTC insertion), infection (intraluminal or systemic), or mechanical (thrombosis, kinking, or dislodgement). In total, 63 STC were removed (94.0%) during the observation period, compared to 40 (83.4%) LTTC (Table 2). Some patients died with the catheter still implanted and, therefore, were not counted as lost accesses. Analysis of intragroup survival revealed a trend for greater STC removal because of infection or mechanical causes, rather than definitive access having been achieved (log-rank; p = 0.051). In the case of the LTTC, removal because of mechanical complications was more frequent than other causes (log-rank; p = 0.002) (Figure 4). No attempts were made to conduct fibrinolysis of catheters by injecting r-TPA or heparin or performing mechanical removal of fibrin from catheter tips.

**Table 2:** Number of cases of removal of non-tunneled short-term catheters (STC) or tunneled long-term catheters (LTTC), by cause. No statistical difference was observed between groups (*Χ* ^2^: 3.108; 2gl; p = 0.21).

Causes of catheter removal	STC		LTTC		
	n	%		n	%
Infection	11	16.4		12	25.0
Mechanical reasons	22	32.8		15	31.3
Definitive access	30	44.8		13	27.1
Total	63	94.0		40	83.4

Catheter-related infections occurred in 57 cases of STC (85.1%) and 34 (70.8%) LTTC (*Χ* ^2^ = 3.4; p = 0.063). Catheter cultures were only performed in ten cases of STC infection and the pathogen was only identified in four cases (three cases of *Staphylococcus sp* and one of multiresistant Acinetobacter), whereas 14 LTTC cases were cultured, with pathogens identified in 6 cases (four cases of *Staphylococcus sp*, one of *Enterobacter cloacae,* and one of *Serratia narcences*). There were insufficient data to study infections of skin or catheter tips, sepsis, or endocarditis. Antibiotic therapy, systemic or intraluminal, as decided by the treating physician, was administered in all cases of catheter infection, irrespective of whether it was removed.

The factors potentially related to occurrence of infection (sex, educational level, healthcare provider, family income, and race) were analyzed individually, and only low income was statistically significant (*X* ^2^ = 35.4; p < 0.0001) (Table 3). The rate of infections per 1,000 days was higher in the STC group than the LTTC group (16.7 events/1,000 days vs. 7.0 events/1,000 days). Accumulated mortality was 210 deaths per 1,000 patients/year for STC and 239 deaths/1,000 patients/year for LTTC.

**Table 3:** Risk factors potentially related to occurrence of catheter infection, irrespective of type.

Risk factors	Infection n (%)	No infection n (%)	p	Test value
Sex				
Male	13 (54)	56 (62)	0.64^†^	na
Female	11 (46)	35 (38)		
Income				
No income	6 (25)	5 (5)		
Up to 1 MW	17 (71)	21 (23)		
(1-5] MW	1 (4)	58 (64)	< 0.0001^£^	35.38
(5-10] MW	0 (0)	6 (7)		
(10-20] MW	0 (0)	1 (1)		

Educational level				
Illiterate	7 (29)	9 (10)	0.10 ^£^	9.075
Primary, incomplete	10 (42)	57 (63)		
Primary, complete	2 (8)	4 (4)		
Secondary, incomplete	0 (0)	3 (3)		
Secondary, complete	2 (8)	12 (13)		
Higher education	3 (13)	6 (7)		

Skin color				
White	18 (75)	69 (76)		
Brown	2 (8)	7 (8)	0.99^£^	0.01
Black	4 (17)	15 (16)		

Healthcare provider				
Public system	22 (92)	86 (95)	0.63^†^	na
Insurance/private	2 (8)	5 (5)		

x MW = multiples of minimum wage; na = not applicable;

^£^Chi-square;

† Fisher’s exact test.

## DISCUSSION

This study, conducted at a hemodialysis service at a teaching hospital, observed that the duration of LTTC was a little more than double the duration of STC (median catheter survival: 50 vs. 112 days) and that low income was the only factor associated with loss of access because of catheter-related infection.

It is estimated that in 2016 there were more than 122 thousand patients on renal substitution therapy in Brazil, which is the equivalent of 596 patients per million members of the population, with the majority (92%) on hemodialysis, which illustrates a gradual increase over the years.^2^ It was also estimated that 20.5% of these patients were using a hemodialysis catheter (approximately 9.4% STC and 11.2% LTTC). This study did not investigate that type of prevalence because it was longitudinal, but our center's internal data indicate that catheter prevalence rates have been around 20 to 30% over the last 3 years (unpublished data).

The general demographic characteristics of the dialysis patients are similar to results in the literature, with a higher prevalence of males (60% of the total).^9^ However, SAH was present as an underlying disease in almost all patients, which contrasts with other Brazilian studies, in which the rate is around 40 to 60%.^5^ ^,^ ^9^ This difference is because, in those studies, SAH was described as the etiology of end-stage kidney disease, rather than a comorbidity present during the study. Notably, the patients fitted with LTTC were approximately 10 years older than those who had an STC fitted, which may indicate that there were difficulties with creation of definitive autologous access in these cases. This age difference has been observed independently in other studies.^10^ ^,^ ^11^

The ideal site for insertion of a central venous catheter is still the subject of debate. Although previous studies have revealed lower rates of infection associated with jugular rather than femoral access, a meta-analysis published in 2012 with data from more than 17 thousand central catheters inserted in hospital settings did not detect evidence of a difference (relative risk [RR] 1.35; 95% CI 0.84-2.19).^12^ Notwithstanding, since hemodialysis catheters are inserted in a specific subset of patients, it is considered that the preference for the jugular access is associated with lower rates of puncture site infections and complications, and a lower incidence of central vein stenosis.^13^ ^,^ ^14^ The chosen side is also essential, since catheters inserted on the right have fewer dysfunctions or infections than those on the left.^15^ In the present study, the right internal jugular vein was used as an access site in more than 80% of cases.

The catheter survival rates observed in this study was lower than in most studies found in the literature, although none have specifically compared STC and LTTC. Mandolfo et al.^16^ observed a cumulative LTTC survival of 91% at 1 year and 85% at 4 years and Shi et al.^17^ observed 82% survival at 1 year and 42% at 4 years. In contrast, Shingarev et al.^14^ reported much lower catheter patency rates, with 54% at 6 months and 35% after 1 year for the right internal jugular vein. In the present study, none of the STC had a duration of more than 156 days and the longest-lasting LTTC survived 338 days. These disparities likely reflect differences in the approach to preservation of accesses, ranging from the socio-economic conditions to interventions for prevention and treatment of infectious and mechanical complications.^18^

In counterpoint, it was not observed differences in overall patient survival over the years, even though the patients in the LTTC group were, on average, 10 years older. A study published in 2018, after observing 140 thousand patients who used hemodialysis catheters (whether as a bridge to an arteriovenous fistula or not), reported similar survival rates to this study but emphasized that mortality and complications were higher in this group of patients.^19^ A Chinese study published in 2017 also observed similar survival.^17^ According to another Brazilian study, the annual gross mortality rate is 18.2%, which is close to the mortality seen in the present study.^2^

In the present study, there were no significant differences between the different causes of loss of the two types of catheteres. The high incidence of mechanical complications observed is notable (thromboses, kinking, and migration) and accounted for a third of both groups. However, in the survival analysis, we observed that the most common cause of LTTC loss was dysfunction of the catheter itself. Incidence of catheter dysfunction was investigated in a Chinese multicenter study with 865 patients that found several factors that were independently associated: rural residence, no anticoagulants, no control imaging exam, a catheter inserted on the left, femoral catheter, and anemia. The same study observed a high incidence of dysfunctions: 66% for STC and 45% for LTTC (p < 0.01).^20^ A prospective cohort study following 154 patients for 16 months observed a 13 times higher incidence of STC dysfunction than tunneled catheters (95% CI 2.9-63.0).^21^

The most common and most serious CVCH complication is infection, which is responsible for a 2 to 3 times greater risk of hospital admissions and death than among patients with an arteriovenous fistula or a prosthetic fistula.^4^ It is associated with a range of risk factors that predispose to elevated morbidity and mortality rates, with an estimated incidence of 60 cases per 10,000 admissions. Infections related to vascular access may be local (infections of the subcutaneous tunnel or the exit site) or systemic (bacteremia and sepsis). Once clinical and local signs of infection have been detected, a blood culture should be taken, parenteral or intraluminal administration of antibiotics should be performed, and catheter removal should be evaluated. If the catheter is removed, a replacement can be inserted, preferably contralaterally, after 48 hours of treatment.^10^

A Canadian multicenter cohort study observed a nine times greater risk of infection with STC and LTTC than with an AVF, without detecting a difference in infections between the two types of catheteres.^22^ It is important that infections are identified not only clinically, but also by microbiology. The present study reported a high incidence of catheter-related infections (85.1% of STC and 70.8% of LTTC), but the rate of catheter tip cultures was negligible (24 out of 91 infection cases, or 26%). Regardless, the rate of infections per patient per year was similar to that in a study published by Sahli et al. (16.6 events/1,000 days),^23^ and higher than rates reported by Murea et al. (1.97/1,000 days of LTTC),^24^ Wang et al. (12.7 events/1,000 catheter days for STC and 5.39 events/1,000 catheter days for LTTC),^20^ and Menegueti et al.^7^ (6.1 bloodstream infections/1,000 dialysis days). A Swedish single-center prospective study observing application of an infection prevention protocol reported colonization, catheter-related infection, catheter-related bloodstream infection rates of 7.0, 2.2, and 0.6 events per 1,000 catheter days, respectively.^18^ Notwithstanding the low prevalence of catheter tip cultures, it was possible to observe a predominance of *S. aureus*, although gram-negative multiresistant strains were also found, in agreement with other Brazilian studies.^25^ ^,^ ^26^

Low income was the only statistically significant variable among those analyzed as potentially related to infection. Ninety-six percent of infection cases had incomes below the minimum monthly wage, whereas 87% of those who did not have an infection had an income between more than one and five times the minimum wage. In common with other studies, it was not possible to relate the occurrence of infections with low educational level, sex, race, or the healthcare provider that paid for treatment.^7^ ^,^ ^22^ It appears that there is an important impact of individual socioeconomic conditions on maintenance of local hygiene, transport difficulties, compliance with medication, purchase of supplies, and other factors.^27^

Negative points of this study include its single-center and retrospective design, the lack of information on the aseptic techniques used when implanting the catheters and on use of medications that potentially change infection rates, and the low number of catheter tip cultures for suspected infection, in part because data collection was dependent on correct completion of the hospital’s patient charts. One result of this is that it was not possible to profile the prevalent flora in catheter-related infection cases adequately. Additionally, the convenience sample with a low number of patients in the LTTC group restricted the inferential power of the comparison of central venous access durability between the two groups studied. No temporal regression analysis could be conducted to better delineate which factors could be associated with loss of the catheter. Finally, the sample's heterogeneous nature must be acknowledged, since different professionals inserted the STC using different methods (with and without ultrasound guidance). The use of ultrasound equipment by the nephrologist, who is the specialist who most often inserts STC at the hemodialysis centers, is rather the exception than the rule in Brazil. This heterogeneity may be another of the study’s positive features since it confers greater external validity.

Reduction of catheter rates and their infectious and mechanical complications can be achieved by implementing local quality programs, with a multidisciplinary team and targets. For example, these programs were able to reduce the catheter rate from 45 to 8% at American dialysis centers treating on Medicare.^28^ It is evident that the possibility of achieving such targets depends on the prevalence of comorbidities (diabetes, for example), age, and the number of prior accesses constructed, which makes creation of durable autologous accesses more difficult.^29^

## CONCLUSIONS

The survival time of long-term tunneled hemodialysis catheters was significantly higher than for short-term catheters but was well below the rates reported in the literature. Regardless, no difference was observed in survival between patients using the different types of catheters. Microbiological analysis of catheter-related infections is still rarely performed, which impacts on their actual incidence. Low income is associated with higher infection rates and is a feature of the scenario at hemodialysis centers in developing countries.
