# Supplementary material for: Analysis of infection rates and duration of short and long-term hemodialysis catheters in a teaching hospital
Source: J Vasc Bras. 2020 Sep 14;19:e20190142. doi: 10.1590/1677-5449.190142 (PMC8276652; doi:10.1590/1677-5449.190142)
Supplement: Translation

# Translation: Análise das taxas de infecção e duração de cateteres de hemodiálise de curta e longa permanência em hospital de ensino

**Authors:** Seleno Glauber de Jesus-Silva, Jennifer dos Santos Oliveira, Karine Tobias França Ramos, Luciene Azevedo Morais, Melissa Andreia de Moraes Silva, Arturo Eduardo Krupa, Rodolfo Souza Cardoso

**Affiliations:** 1 Associação de Integração Social de Itajubá – AISI, Hospital de Clínicas de Itajubá – HCI, Departamento de Cirurgia Vascular e Endovascular, Itajubá, MG, Brasil.; 2 Faculdade de Medicina de Itajubá – FMIt, Itajubá, MG, Brasil.; 3 Associação de Integração Social de Itajubá – AISI, Hospital de Clínicas de Itajubá – HCI, Departamento de Nefrologia, Itajubá, MG, Brasil.

**Keywords:** diálise renal, infecções relacionadas a cateter, cateteres de demora

## Abstract

**Contexto:**

Os cateteres venosos centrais para hemodiálise (CVCH) de curta permanência (CCP) e cateteres tunelizáveis de longa permanência (CTLP) são fundamentais para a terapia hemodialítica. Entretanto, há escassa casuística nacional acerca da incidência de complicações desses dois tipos de cateteres.

**Objetivos:**

Analisar as complicações e tempo de duração de CVCHs em centro de hemodiálise de hospital de ensino.

**Métodos:**

Foi feito um estudo unicêntrico, longitudinal e retrospectivo de 115 pacientes consecutivos submetidos a implante de cateteres para hemodiálise (67 CCP e 48 CTLP) em um período de 2 anos, com análise de sobrevida geral, perviedade, perda do acesso e incidência de complicações.

**Resultados:**

Sessenta por cento eram do sexo masculino e a média de idade foi de 62 anos. O principal sítio de punção foi a veia jugular interna direita. Hipertensão arterial sistêmica estava presente em 95% dos casos. A mediana de permanência do cateter foi de 50 dias (CCP) *versus* 112 dias (CTLP; p < 0,0001). Não houve diferença na sobrevida global. Infecção relacionada ao cateter apresentou maior incidência nos CCP, sendo *Staphylococcus sp.* o microrganismo mais encontrado. A taxa de infecção por 1.000 dias foi maior nos CCP em relação aos CTLP (16,7 eventos/1.000 dias *versus* 7,0 eventos/1.000 dias, respectivamente). Baixa renda foi o único fator relacionado a maior incidência de infecção.

**Conclusões:**

O tempo de permanência dos CTLP foi significativamente maior que os CCP, porém ainda assim abaixo dos valores relatados na literatura e sem impacto na sobrevida global. Baixa renda foi um fator associado a infecção de cateter.

## INTRODUÇÃO

A hemodiálise é um recurso amplamente utilizado no tratamento da insuficiência renal crônica terminal e envolve vários aspectos críticos para a sua execução, como o acesso vascular. Apesar de ser uma modalidade que, em geral, mantém a qualidade de vida do paciente, ela carrega consigo altos índices de complicação e internações, com taxa de mortalidade chegando a 10 a 25% por ano^1^. Embora o acesso ideal para a manutenção de hemodiálise a longo prazo seja a fístula arteriovenosa (FAV) ou a fístula com prótese, a realidade no Brasil é o uso, por grande parte dos pacientes, de cateteres venosos centrais para hemodiálise (CVCH), com uma prevalência de até 20,5%^2^. O uso de CVCHs tem aumentado, ao mesmo tempo em que a população envelhece e mais pacientes com poucas opções de confecção de FAV iniciam a hemodiálise.

Os cateteres não tunelizáveis de curta permanência (CCP) representam uma opção confiável nas situações que requerem início imediato à terapia de substituição renal e que não possuam acesso definitivo, mas que devem ser removidos o mais rápido possível, devido ao alto risco de complicações infecciosas^3^. Nos casos de impossibilidade de confecção de uma FAV, seja por ausência de veia adequada ou por debilidade clínica, os cateteres tunelizáveis de longa permanência (CTLP) são opções consideradas mais duradouras e com menores complicações^4^. O uso de CCP por mais de 3 meses é indicador negativo de qualidade dos serviços de hemodiálise no Brasil^5^.

Diversas complicações estão relacionadas aos CVCHs, incluindo as de implantação (hematomas, pseudoaneurismas, pneumotóraces), tromboses do cateter e de veias centrais, além da mais grave de todas, a infecção relacionada ao cateter. Esta última é associada a elevadas taxas de internação, custo e mortalidade^6^. Diversos fatores foram associados à perda dos CVCHs, como idade avançada, história de múltiplos acessos prévios, grau de escolaridade, qualidade da manipulação do dispositivo pela equipe de enfermagem, diabetes, imunodepressão, entre outros^7^ ^,^ ^8^.

Apesar do grande conhecimento disponível na literatura estrangeira acerca da evolução dos CVCHs, poucos estudos regionais abordaram comparativamente suas taxas de permanência e de complicações. São necessárias, portanto, casuísticas que definam se os resultados de vida real em centro de hemodiálise brasileiros são comparáveis aos previstos na literatura, de forma a diagnosticar e corrigir possíveis falhas. O presente estudo teve por objetivo analisar, em centro de hemodiálise de hospital de ensino, as diferentes evoluções dos CCP e CTLP no que diz respeito a durabilidade, complicações infecciosas e não-infecciosas e fatores de risco associados.

## MÉTODOS

Foi feito um estudo retrospectivo, observacional e longitudinal, no qual foram analisados, por meio de revisão de prontuários, 115 pacientes consecutivos submetidos a implante de CVCH (67 CCP e 48 CTLP) em serviço de terapia renal substitutiva de hospital de ensino quaternário, no período de janeiro de 2016 a janeiro de 2018. O estudo foi aprovado pelo Comitê de Ética em Pesquisa da instituição sob parecer n^o^ 2.170.323.

O cálculo da amostra foi baseado na diferença estimada de permanência do CVCH em 30 dias (60% para CCP e 90% para CTLP), poder de teste de 80% e nível de significância de 95%, totalizando-se 69 indivíduos por grupo. Como não foi possível alcançar a quantidade suficiente de indivíduos no grupo CTLP para o período estudado, a amostra foi considerada de conveniência.

Os CCP foram definidos como cateteres venosos centrais de hemodiálise não tunelizáveis, independentemente do tempo de duração (modelos utilizados: Arrow-Howes®LargeBore 12 Fr 16 e 20 cm, Teleflex, Morrisville, Carolina do Norte, EUA; e Duo-Flow®Side x Side 12 Fr 15 e 20 cm, Medcomp, Harleysville, Pensilvânia, EUA). Os CTLP foram definidos como cateteres tunelizáveis de duplo lúmen (modelo Hemo-Cath® LT 12,5 Fr 28 e 32 cm, Medcomp). Os CCP foram inseridos por nefrologista ou por cirurgião vascular em sala de procedimento sem auxílio de fluoroscopia. Somente os CCP implantados pelo cirurgião tiveram orientação ultrassonográfica, enquanto os nefrologistas utilizaram técnica de marcação anatômica convencional. Todos os CTLP foram implantados por cirurgião vascular em sala de hemodinâmica, por meio de técnica convencional e asséptica sob orientação ultrassonográfica. Não foi administrado nenhum antimicrobiano no peroperatório para prevenção de infecção do acesso, conforme protocolo institucional. Entretanto, todos os pacientes faziam uso ao menos de antiagregação plaquetária (ácido acetilsalicílico 100 mg/dia ou bissulfato de clopidogrel 75 mg/dia) ou anticoagulação oral. Não foi possível rastrear possíveis falhas de aderência no uso dos antiagregantes ou anticoagulantes ao longo do acompanhamento. Os dados foram extraídos a partir de banco de dados dedicado ao serviço de hemodiálise (NefroSys®, Porto Alegre, RS) e inseridos em planilha eletrônica. A alimentação do banco de dados eletrônico foi feita de forma contínua por equipe multiprofissional. Foram incluídos todos os pacientes submetidos ao implante de cateter de curta ou longa permanência que apresentaram prontuários completos e excluídos aqueles com cateteres implantados em veia femoral. Somente o primeiro implante de cada tipo de cateter foi considerado para estudo. Pacientes cujos CCP foram substituídos por CTLP ao longo do estudo foram incluídos na análise de duração do cateter duas vezes. Para a análise de sobrevida global, somente os casos em que os CCP se tornaram o único tipo de acesso utilizado foram incluídos. As falhas técnicas relacionadas aos implantes dos cateteres que não permitiram o seu uso não foram contabilizadas como acesso realizado e, consequentemente, eles não foram elegíveis a ser incluídos no estudo.

Foram coletadas informações epidemiológicas (idade, sexo, raça, escolaridade, renda familiar, tipo de provedor de saúde), data de implante e retirada do cateter, motivo de retirada, complicações, fatores de risco presentes [hipertensão arterial sistêmica (HAS), diabetes melito, dislipidemia, tabagismo], presença de infeção e patógeno identificado e data de óbito. HAS foi considerada como pressão arterial maior que 140 × 90 mm Hg ou uso de hipotensivos, diabetes melito como glicemia de jejum > 126 mg/dL ou glicemia pós-prandial > 200 mg/dL ou uso contínuo de hipoglicemiantes, dislipidemia como LDL > 130 mg/dL ou uso de estatina, e tabagismo como qualquer uso contínuo de pelo menos um cigarro por dia. Os dados de cultura aeróbia foram utilizados por meio da ponta do cateter, seccionada no momento da retirada em via estéril, e o resultado foi dado como positivo se > 100 UFC/mm^2^ fossem isoladas.

A estatística descritiva foi dada pela média ou mediana e porcentagem. A estatística inferencial foi dada pelo teste *t* para amostras independentes bicaudal, teste de Qui-quadrado (*X* ^2^) ou teste de Fisher, dependendo das variáveis analisadas, após a identificação de *outliers* pelo método ROUT. A comparação da taxa de duração do cateter foi realizada pela curva de sobrevida pelo método de Kaplan-Meier, com cálculo do *log-rank*. Foi calculada a taxa global de infecção por 1.000 dias de cateter e a taxa de mortalidade por 1.000 pacientes/ano. Foi utilizado o *software* estatístico GraphPad Prism v.8, com significância estatística se p < 0,05 e índice de confiança de 95%.

## RESULTADOS

Cento e quinze pacientes foram analisados, sendo que 67 foram submetidos ao implante de CCP e 48, de CTLP (Figura 1). Sessenta por cento eram do sexo masculino e a média de idade foi de 62 anos. A veia jugular interna direita foi o principal sítio de punção, tendo sido usada em 85,1% dos CCP e em 79,2% dos CTLP. Houve uma prevalência muito grande de HAS (95,7%) e diabetes melito (47%), enquanto dislipidemia (23,5%) e tabagismo (12,2%) foram menos frequentes. Não houve diferença quanto ao índice de massa corporal (IMC) em nenhum dos grupos. Dos 67 casos de CCP, 20 (29,9%) foram inseridos por cirurgião vascular. A Tabela 1 mostra as características clínicas e frequência dos sítios de acesso utilizados em ambos os grupos. Todos os pacientes foram acompanhados neste estudo até o momento da retirada do cateter ou do óbito.

**Figura 1:** Diagrama de fluxo dos pacientes inseridos no estudo. CCP = cateteres não tunelizáveis de curta permanência; CTLP = cateteres tunelizáveis de longa permanência.

**Tabela 1:** Características epidemiológicas e fatores de risco presentes na amostra e em cada um dos subgrupos analisados.

Fatores de risco	Total n (%)	CCP n (%)	CTLP n (%)	p
Idade (anos)	62,2 (±15,0)^*^	58,2 (±14,5)^*^	67,8 (±13,9)^*^	0,0006^§^
Sexo				
Masculino	69 (60)	44 (63,8)	25 (36,2)	0,14^£^
Feminino	46 (40)	23 (50)	23 (50)	
IMC	24 (±5,6)^*^	24,2 (±5,8)^*^	23,7 (±5,4)^*^	0,69^§^
Comorbidades				
Hipertensão	110 (95,7)	63 (94)	47 (98)	0,39 ^†^
Diabetes melito	54 (47)	30 (45)	24 (50)	0,58^£^
Dislipidemia	27 (23,5)	9 (13,4)	18 (37,5)	0,003^†^
Tabagismo	14 (12,2)	11 (16,4)	3 (6,3)	0,14^†^
Tipo de acesso				0,18^£^
Femoral	4 (3,5)	3 (4,5)	1 (2,1)	
Jugular D	95 (82,6)	57 (85,1)	38 (79,2)	
Jugular E	14 (12,2)	5 (7,5)	9 (18,8)	
VAx	2 (1,7)	2 (3,0)	0 (0)	

CCP = cateter não tunelizável de curta permanência; CTLP = cateter tunelizável de longa permanência; IMC = índice de massa corporal; VAx = veia axilar;

* desvio-padrão;

§ teste *t* de Student;

^£^Qui-quadrado;

† teste exato de Fisher.

O tempo de permanência dos cateteres foi significativamente diferente, sendo que os CCP tiveram uma mediana de 50 dias e os CTLP, de 112 dias [IC (intervalo de confiança) 95%, CCP: 45,1-63,3 dias *versus* CTLP: 101,7-159,7 dias; *log-rank*, p < 0,0001] (Figura 2). Houve 21 óbitos entre os usuários de CCP e 20 entre os de CTLP durante todo o período de acompanhamento. A mortalidade operatória (30 dias) foi de 1,5% para o grupo CCP, enquanto não foi observado óbito no grupo CTLP no período mencionado. Em relação à mortalidade global, a análise de sobrevida não identificou diferença entre os grupos (mediana de sobrevida CCP: 2,87 anos *versus* CTLP: 3,34 anos; *log-rank*, p = 0,68), sendo observada uma mortalidade bruta no primeiro ano de 20% para os CCP e 23,5% para os CTLP (Figura 3).

**Figura 2:** Curva de sobrevida de Kaplan-Meier do tempo de permanência entre os cateteres não tunelizáveis de curta permanência (CCP) e os cateteres tunelizáveis de longa permanência (CTLP), implantados ao longo de um período máximo de 338 dias de observação. Foi observada diferença significativa entre os grupos (teste *log-rank*).

**Figura 3:** Curva de Kaplan-Meier para a sobrevida global dos pacientes submetidos ao implante de cateteres de curta permanência (CCP) e de cateteres tunelizáveis de longa permanência (CTLP), ao longo de 4,8 anos de observação. Não foi observada diferença significativa entre os grupos (teste *log-rank*).

As causas de retirada do cateter foram classificadas como obtenção de acesso definitivo (confecção de fístula arteriovenosa ou implante de CTLP), infecção (intraluminal ou sistêmica) e causa mecânica (trombose, acotovelamento ou exteriorização). No total, 63 CCP foram retirados (94,0%), enquanto 40 (83,4%) dos CTLP foram removidos no período de observação (Tabela 2). Alguns pacientes foram a óbito com o cateter implantado; portanto, não foram incluídos como perda do acesso. A análise de sobrevida intragrupo revelou uma maior tendência de retirada dos CCP nos casos de infecção ou causa mecânica em detrimento da obtenção de acesso definitivo (*log-rank*; p = 0,051). No caso dos CTLP, a principal causa de retirada foi complicação mecânica em relação às demais (*log-rank*; p = 0,002) (Figura 4). Não foi realizada nenhuma tentativa de fibrinólise do CVCH por meio de injeção de r-TPA ou heparina, ou de remoção mecânica de fibrina em ponta de cateter.

**Tabela 2:** Número da casos de retirada de cateteres não tunelizáveis de curta permanência (CCP) ou cateteres tunelizáveis de longa permanência (CTLP) conforme a causa. Não foi observada diferença estatística entre os grupos (*Χ* ^2^: 3,108; 2gl; p = 0,21).

Causas de retirada de cateter	CCP		CTLP	
	n	%	n	%
Infecção	11	16,4	12	25,0
Causas mecânicas	22	32,8	15	31,3
Acesso definitivo	30	44,8	13	27,1
Total	63	94,0	40	83,4

**Figura 4:** Curva de Kaplan-Meier do tempo de permanência dos cateteres em relação ao motivo de retirada [infecção do cateter; realização de acesso definitivo, seja fístula arteriovenosa ou implante de cateter tunelizável de longa permanência (CTLP); ou causas mecânicas como trombose, acotovelamento ou exteriorização]. No grupo CCP (cateter não tunelizável de curta permanência), não foi observada diferença significativa, porém o implante de acesso definitivo foi a causa menos comum de retirada. No grupo CTLP, as causas mecânicas foram o principal motivo de retirada ou troca do cateter.

A infecção relacionada ao cateter ocorreu em 57 casos de CCP (85,1%) e em 34 (70,8%) CTLP (*X* ^2^ = 3,4; p = 0,063). Somente dez casos de infecção em CCP foram submetidos à cultura de cateter, sendo identificado o patógeno em somente quatro casos (três *Staphylococcus sp* e um *Acinetobacter* multirresistente), enquanto nos CTLP, 14 foram submetidos à cultura, com identificação de 6 casos (quatro *Staphylococcus sp*, um *Enterobacter cloacae* e uma *Serratia narcences*). Não houve dados suficientes para estudo sobre infecção de pele, de ponta de cateter, sepse ou endocardite. Em todos os casos de infecção de cateter, submetidos ou não à retirada, foi administrada antibioticoterapia sistêmica ou intraluminal, a critério do médico assistente.

Ao analisar individualmente os diferentes fatores potencialmente relacionados à ocorrência de infecção (sexo, escolaridade, convênio médico, renda familiar e raça), somente a baixa renda apresentou resultado estatisticamente significante (*X* ^2^ = 35,4; p < 0,0001) (Tabela 3). A taxa de infecção por 1.000 dias foi maior nos CCP em relação aos CTLP (16,7 eventos/1.000 dias *versus* 7,0 eventos/1.000 dias). A mortalidade acumulada foi de 210 óbitos por 1.000 pacientes/ano para os CCP e 239 óbitos/1.000 pacientes/ano para os CTLP.

**Tabela 3:** Fatores de risco potencialmente relacionados à ocorrência de infecção de cateter, independentemente do tipo.

Fatores de Risco	Infecção n (%)	Sem Infecção n (%)	p	Valor do teste
Sexo				
Masculino	13 (54)	56 (62)	0,64^†^	na
Feminino	11 (46)	35 (38)		
Renda				
Sem renda	6 (25)	5 (5)		
Até 1 SM	17 (71)	21 (23)		
(1-5] SM	1 (4)	58 (64)	< 0,0001^£^	35,38
(5-10] SM	0 (0)	6 (7)		
(10-20] SM	0 (0)	1 (1)		

Escolaridade				
Analfabeto	7 (29)	9 (10)	0,10 ^£^	9,075
1º grau incompleto	10 (42)	57 (63)		
1º grau completo	2 (8)	4 (4)		
2º grau incompleto	0 (0)	3 (3)		
2º grau completo	2 (8)	12 (13)		
Superior	3 (13)	6 (7)		

Raça/Etnia				
Branco	18 (75)	69 (76)		
Pardo	2 (8)	7 (8)	0,99 ^£^	0,01
Negro	4 (17)	15 (16)		

Provedor				
Sistema público	22 (92)	86 (95)	0,63 ^†^	na
Convênio/privado	2 (8)	5 (5)		

SM = salário mínimo; na = não aplicável;

^£^Qui-quadrado;

† teste exato de Fisher.

## DISCUSSÃO

Este trabalho observou um tempo de duração dos CTLP correspondente a pouco mais que dobro dos CCP (mediana de sobrevida de 50 *versus* 112 dias) e baixa renda como fator associado à perda do acesso devido à infeção relacionada ao cateter, em serviço de hemodiálise de hospital de ensino.

Estima-se que, no Brasil, no ano de 2016, havia mais de 122 mil pacientes em terapia de substituição renal, o equivalente a uma taxa de 596 pacientes por milhão da população, estando a maioria (92%) em hemodiálise, o que indica um aumento gradual ao longo dos anos^2^. Estimou-se que 20,5% desses pacientes faziam uso de cateter de hemodiálise (aprox. 9,4% CCP e 11,2% CTLP). O presente trabalho não estudou esse tipo de prevalência por ser longitudinal; entretanto, dados internos do serviço apontam para prevalências de cateteres em torno de 20 a 30% ao longo dos últimos 3 anos (não publicados).

As características demográficas gerais dos pacientes dialíticos apresentaram resultados semelhantes à literatura, com maior prevalência do sexo masculino (60% do total)^9^. A HAS, entretanto, estava presente na quase totalidade dos pacientes como doença de base, o que contrasta com outros estudos nacionais, em que essa taxa gira em torno de 40 a 60%^5^ ^,^ ^9^. Essa diferença ocorreu uma vez que a HAS nesses trabalhos foi descrita como etiologia para a doença renal terminal, e não como comorbidade presente ao longo do estudo. Interessante notar que os pacientes submetidos ao implante de CTLP se encontravam em faixa etária aproximadamente 10 anos maior que os de CCP, o que pode indicar a dificuldade na confecção de um acesso autólogo definitivo nesses casos. Essa discrepância de idade já foi observada isoladamente em outros estudos^10^ ^,^ ^11^.

O sítio ideal de implante do cateter venoso central ainda é tema de discussão. Embora estudos anteriores tenham mostrado uma menor taxa de infeção relacionada ao acesso jugular em relação ao femoral, uma metanálise publicada em 2012 com mais de 17 mil cateteres centrais simples implantados em ambiente hospitalar não evidenciou diferença [risco relativo (RR) 1,35; IC 95% 0,84-2,19]^12^. Apesar disso, entende-se que, em relação aos cateteres de hemodiálise, devido ao subgrupo específico de usuários, a preferência pelo acesso jugular está associada a menor infeção e complicações de punção, além de menor incidência de estenose de veia central^13^ ^,^ ^14^. A lateralidade também é importante, uma vez que cateteres implantados à direita possuem menos disfunção ou infecção do que à esquerda^15^. No presente trabalho, a prevalência de uso de veia jugular interna direita como sítio de acesso ocorreu em mais de 80% dos casos.

As taxas de sobrevida de cateter observadas neste estudo estão abaixo da maioria dos trabalhos encontrados na literatura, apesar de não haver estudo que compare especificamente os CCP e CTLP. Mandolfo et al.^16^ encontraram uma sobrevida cumulativa de CTLP de 91% em 1 ano e de 85% em 4 anos, enquanto Shi et al.^17^ observaram sobrevida de 82% em 1 ano e de 42% em 4 anos. Por outro lado, Shingarev et al.^14^ descreveram taxas de perviedade de cateteres bem menores, de 54% em 6 meses e 35% em 1 ano para veia jugular interna direita. No presente trabalho, não houve CCP com duração maior que 156 dias ou CTLP maior que 338 dias. Essa disparidade deve refletir diferentes realidades na abordagem de preservação dos acessos, desde condições socioeconômicas até intervenções para prevenção e tratamento de complicações infecciosas e mecânicas^18^.

Por outro lado, não foi observada diferença na sobrevida global dos pacientes ao longo dos anos, mesmo que os pacientes do grupo dos CTLP fossem, em média, 10 anos mais velhos. Um estudo publicado em 2018, após observação de 140 mil pacientes usuários de cateter de hemodiálise (como ponte ou não para fístula arteriovenosa), observou sobrevida semelhante ao presente trabalho, porém ressaltou uma maior mortalidade e complicações nesse grupo de pacientes^19^. Um estudo chinês publicado em 2017 também observou sobrevida semelhante^17^. Segundo um estudo brasileiro, a taxa de mortalidade bruta anual é de 18,2%, o que demonstra uma proximidade com a mortalidade do presente estudo^2^.

No presente trabalho, não houve diferenças significativas entre as diferentes causas estudadas de perda de cateter para ambos os tipos. Deve-se ressaltar a alta incidência de complicações mecânicas observadas (tromboses, acotovelamentos, migrações etc.), chegando a um terço dos casos em ambos os grupos. Na análise de sobrevida, entretanto, observamos que a principal causa de perda do CTLP foi justamente a disfunção do cateter. A incidência de disfunção de cateter foi publicada em estudo chinês multicêntrico com 865 pacientes, sendo observados vários fatores independentemente relacionados: residência rural, ausência de anticoagulantes, ausência de exame de imagem de controle, cateter implantado à esquerda, cateter femoral e anemia. No mesmo estudo, observou-se uma alta incidência de disfunção: 66% para CCP e 45% para CTLP (p < 0,01)^20^. Uma coorte prospectiva envolvendo 154 pacientes durante 16 meses de estudo observou uma incidência 13 vezes maior de disfunção de CCP em comparação com os cateteres tunelizáveis (IC 95% 2,9-63,0)^21^.

A complicação dos CVCH mais relatada e mais temida é a infecção, a qual gera um risco de hospitalização e morte de 2 a 3 vezes maior que os portadores de fistula arteriovenosa ou fístulas protéticas^4^. É associada a diversos fatores de risco que predispõem à elevada taxa de morbimortalidade dos pacientes, com uma incidência estimada de 60 casos por 10.000 admissões. As infecções relacionadas ao acesso vascular podem ser tanto locais (infecções de óstio ou de túnel) como sistêmicas (bacteremias e sepses). Uma vez apresentando sinais clínicos e/ou locais de infecção, deve-se realizar a hemocultura e a administração de antibiótico parenteral ou intraluminal e ser avaliada a remoção cateter. Caso seja removido o cateter, um novo poderá ser inserido, preferencialmente no lado contralateral, após 48 h de terapia^10^.

Uma coorte multicêntrica canadense, entretanto, observou um risco 9 vezes maior de infecção de CCP e CTLP em relação às FAV, sem notar diferença nas infecções entre os dois tipos de cateteres^22^. É importante que sejam identificadas não somente as infecções sob o aspecto clínico, mas também microbiológico. O presente trabalho revelou alta incidência de infecção relacionada ao cateter (85,1% nos CCP e 70,8% nos CTLP), porém a taxa de cultura de ponta do cateter foi desprezível (24 de 91 casos de infecção, ou 26%). Apesar disso, a taxa de infeção por paciente por ano relatada foi semelhante à do estudo publicado por Sahli et al. (16,6 eventos/1.000 dias)^23^, e superior à de Murea et al. (1,97/1.000 dias de CTLP)^24^, de Wang et al. (12,7 eventos/1.000 dias de cateteres para os CCP e 5,39 eventos/1.000 dias de cateteres para os CTLP)^20^ e de Menegueti et al.^7^ (6,1 infecções de corrente sanguínea/1.000 dias de diálise). Um estudo prospectivo unicêntrico sueco, aplicando protocolo de prevenção de infecção, obteve incidências de colonização, infecção relacionada ao cateter e infecção de corrente sanguínea relacionado ao cateter de 7,0, 2,2 e 0,6 eventos por 1.000 dias de cateteres^18^. Apesar da baixa prevalência de culturas de ponta de cateter, foi possível observar a predominância do *S. aureus*, porém com ressalva para ocorrência de gram-negativos multirresistentes, em concordância com outros estudos nacionais^25^ ^,^ ^26^.

Entre os fatores estudados potencialmente relacionados à ocorrência de infecção, somente a baixa renda foi estatisticamente relacionada. Noventa e seis por cento dos que apresentaram infecção possuíam até um salário mínimo de renda, enquanto 87% dos que não apresentaram infecção recebiam de um até cinco salários mínimos. Semelhante a outros estudos, não foi possível relacionar a ocorrência de infecção com baixa escolaridade, sexo, raça ou provedor do tratamento^7^ ^,^ ^22^. Parece ser importante o impacto que as condições socioeconômicas individuais devem ter na preservação da higiene local, dificuldade de transporte, aderência às medicações, compra de materiais, entre outras^27^.

Entre os pontos negativos do trabalho, podemos enumerar a característica unicêntrica e retrospectiva, a ausência de informações referentes às técnicas de assepsia para implantação dos cateteres, o uso de medicações que alterem o índice de infecção, além da baixa incidência de cultura de ponta de cateter realizada nas suspeitas de infecção, em parte porque a coleta de dados dependeu do correto preenchimento dos prontuários hospitalares. Devido a isso, não foi possível a adequada caracterização da flora prevalente nos casos de infecção relacionada ao cateter. Ainda, a amostra por conveniência e com número reduzido no grupo CTLP não permitiu poder de inferência na comparação de durabilidade do acesso venoso central entre os grupos estudados e não foi realizada análise de regressão temporal para definir melhor quais fatores poderiam estar associados à perda do cateter. Por fim, deve-se reconhecer a heterogeneidade da amostra, na qual profissionais distintos realizaram o implante de CCP sob diferentes métodos (com e sem orientação ultrassonográfica). O uso do equipamento de ultrassonografia por parte do nefrologista, sendo este o especialista que mais executa o implante do CCP nos centros de hemodiálise, não é uma realidade no Brasil. Tal heterogeneidade pode ser um ponto positivo do estudo, entretanto, pois confere maior validação externa dos resultados.

A diminuição das taxas de cateteres e suas complicações infecciosas e mecânicas podem ser alcançadas com a instalação de programas locais de qualidade, com equipe multidisciplinar e metas a serem alcançadas. Tais programas, por exemplo, possibilitaram uma redução na taxa de cateter de 45 para 8% em centros americanos de diálise que utilizam o *Medicare* ^28^. É evidente que a possibilidade de alcançar tais metas depende também da prevalência de comorbidades (diabetes, por exemplo), idade e quantidade de acessos prévios criados, o que dificulta a confecção de acessos autólogos de alta durabilidade^29^.

## CONCLUSÃO

O tempo de sobrevida dos cateteres de hemodiálise tunelizáveis de longa permanência foi significativamente maior que os de curta permanência, porém ainda assim abaixo dos valores relatados na literatura. Apesar disso, não foi observada diferença de sobrevida dos pacientes utilizando qualquer um dos tipos de cateteres. A análise microbiológica das infecções relacionadas a cateter ainda é pouco realizada, o que impacta na sua real incidência. A baixa renda esteve associada a maior taxa de infecção, o que reflete a realidade de um serviço de hemodiálise de país em desenvolvimento.
